# Supplementary material for: A column-like organization for ocular dominance in mouse visual cortex
Source: Nat Commun. 2025 Feb 25;16:1926. doi: 10.1038/s41467-025-56780-3 (PMC11861588; doi:10.1038/s41467-025-56780-3)
Supplement: Supplementary file 1 — Supplementary Information [file 41467_2025_56780_MOESM1_ESM.pdf]

Supplementary information for

A column-like organization for ocular dominance in mouse visual cortex

Goltstein, P.M., Laubender, D., Bonhoeffer, T., & Hübener, M.

Includes:

Supplementary Figures 1 – 14

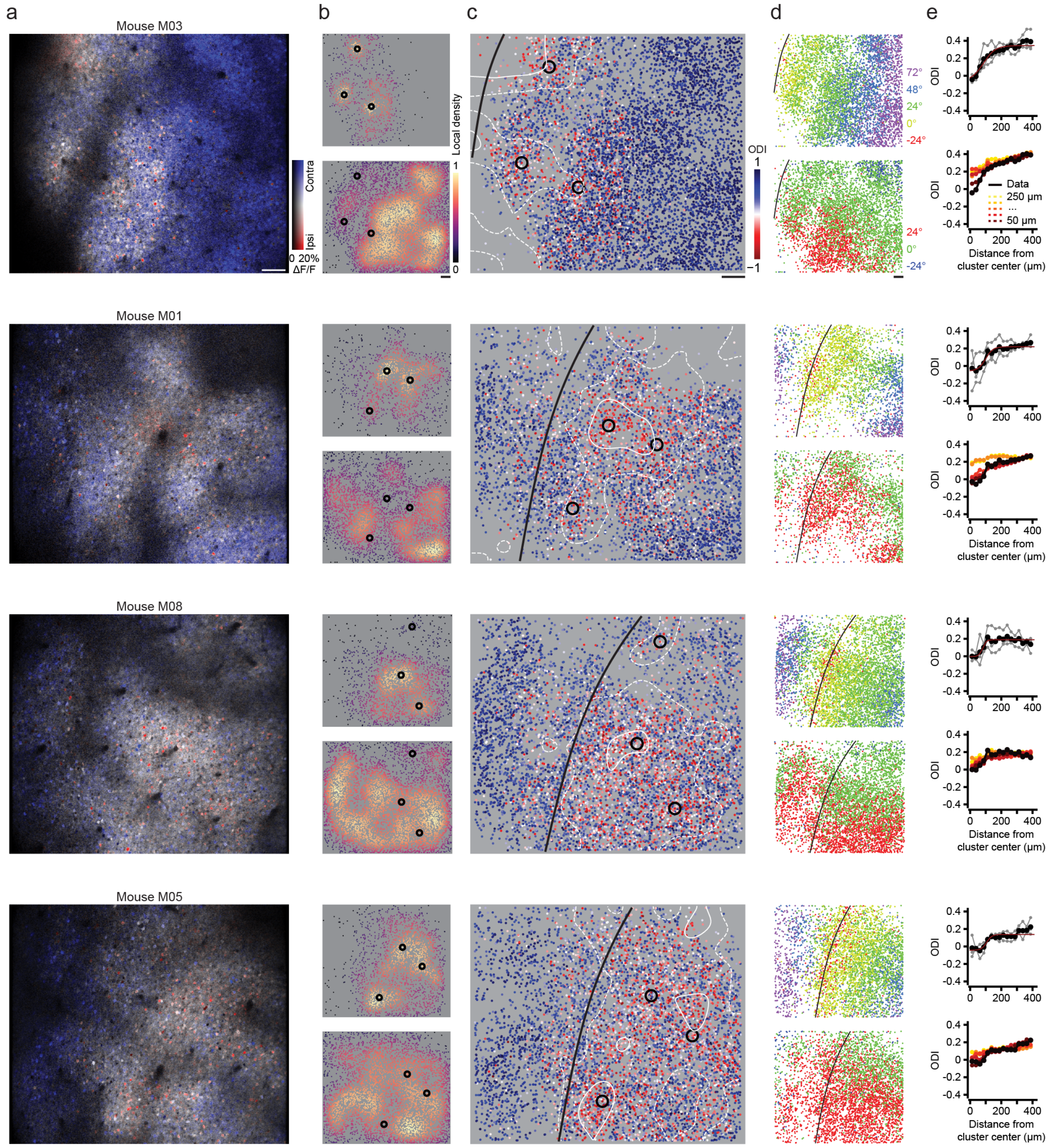

Supplementary Figure 1 | Examples of ipsilateral eye preferring ocular dominance clusters in GCaMP6s transgenic mice.

Each row shows data of all visually responsive neurons recorded from four imaging planes in layer 4 of a single mouse (see Fig. 1a). **a,** HLS map for ocular dominance. Hue: Eye-preference (contralateral: Blue; ipsilateral: Red). Lightness: ΔF/F response amplitude. Saturation: Eye selectivity). **b,** Local density for each visually responsive neuron. Top: Ipsilateral eye preferring neurons. Bottom: Contralateral eye preferring neurons. Black circles mark the centers of ipsi-clusters, detected in the local density map for ipsilateral eye preferring neurons (top). **c,** Ocular dominance of each visually responsive neuron. White iso-ODI lines delineate ODI = 0 (solid) and ODI = 0.2 (dashed), the black line shows the lateral border of V1 (see **d**). **d,** Preferred azimuth (top) and elevation (bottom) in the layer 4 volume. Black line: V1 lateral boundary. **a-d,** Scale bar: 200 µm. **e,** Top: ODI as a function of distance to ipsi-cluster centers (Gray: Individual ipsi-clusters. Black: Mean across ipsi-clusters). Bottom: As top, but for the local randomization control, swapping the ODIs of pairs of cells at a radius of 50 µm (dark red) to 250 µm (yellow).

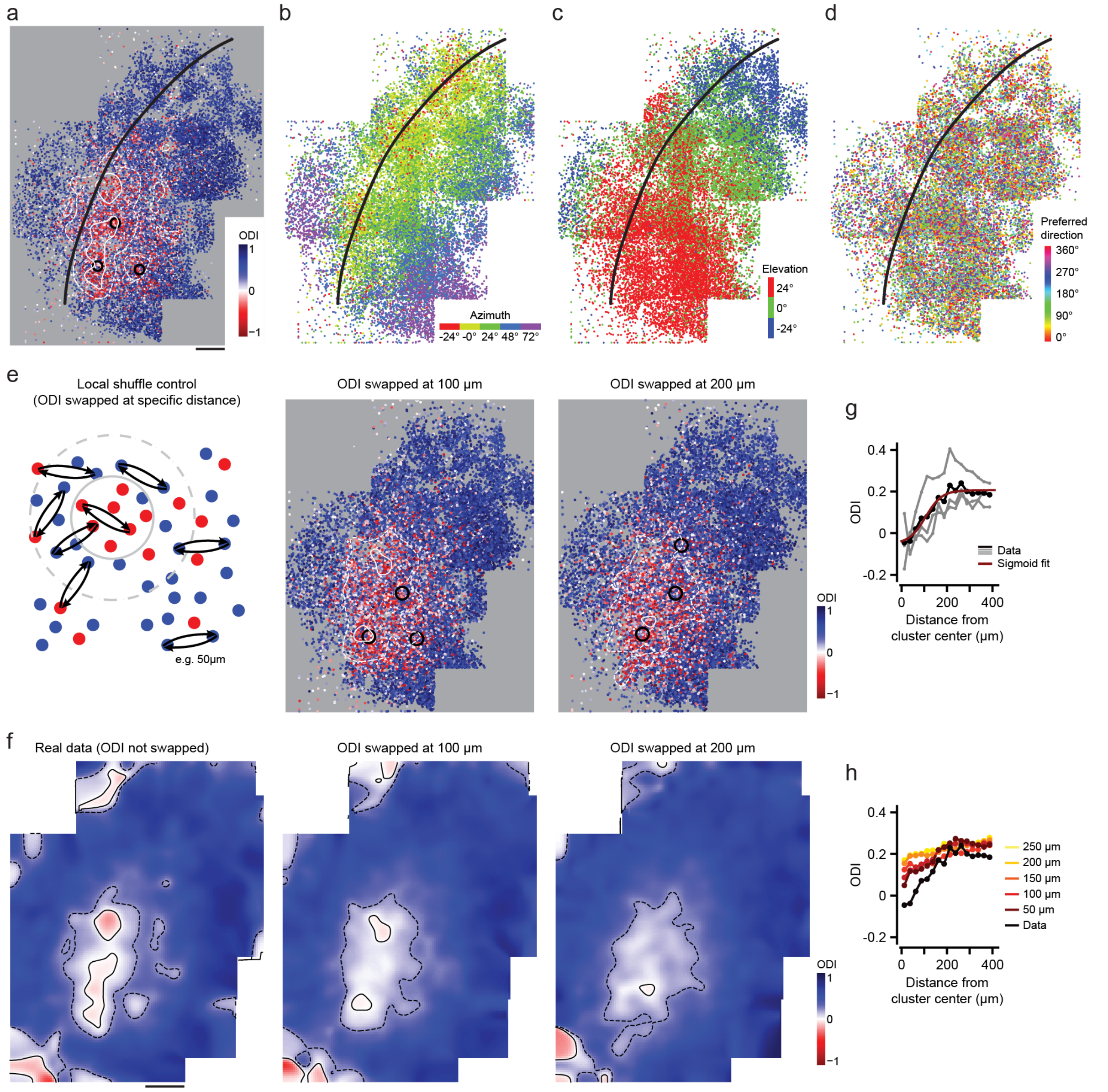

Supplementary Figure 2 | Tiled, large-field of view feature maps of mouse binocular visual cortex and the effect of local randomization by repositioning cells within a fixed radius of their original position.
a-d, Spatial position of all visually responsive neurons from five multiplane layer 4 recordings, stitched into a single map color-coded for **a**, eye preference, **b**, preferred azimuth, **c**, preferred elevation and **d**, preferred orientation (data of mouse M06). Black line: Estimated boundary separating primary visual cortex (right) and lateral higher visual areas (left). White lines in (**a**) indicate ODI = 0 (solid) and ODI = 0.2 (dashed), black circles mark identified ipsi-clusters. Scale bar: 200 µm. **e,** Left: Schematic illustrating the process of local randomization by swapping the ODIs of pairs of neurons at a specified distance. Middle: ODI map following local randomization at a distance of 100 µm. Right: As middle, but for swapping pairs of neurons at 200 µm distance. White: Iso-ODI lines for ODI = 0 (solid) and ODI = 0.2 (dashed). Black circles: Centers of ipsi-clusters detected in each data set. **f,** Smoothed ODI maps, based on the data shown in (**a**) and (**e**). Smoothing was done using a 50 µm Gaussian kernel. Black: Iso-ODI lines for ODI = 0 (solid) and ODI = 0.2 (dashed). Scale bar: 200 µm. **g,** ODI as a function of distance to ipsi-cluster centers for the data in (**a**), left. Gray: Data for individual ipsi-cluster centers. Black: Mean. Red: Sigmoid fit. **h,** As (**g**), but showing the mean ODI "In" ipsi-clusters for different distances at which the ODIs of neuron-pairs were swapped (local randomization control, 100 swaps per distance). Ipsi-clusters were detected anew for each local randomization individually.

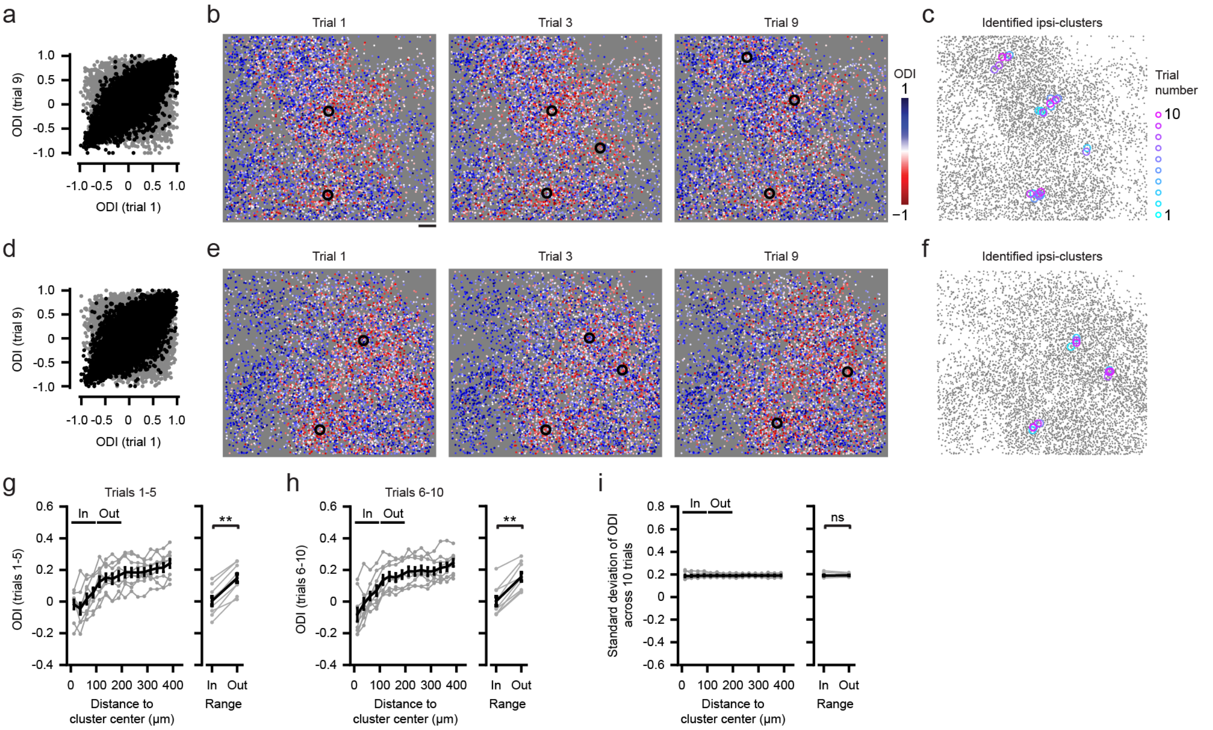

Supplementary Figure 3 | Trial-to-trial variability in ODI and ipsi-clusters

a, Scatter plot of single neuron ODI values calculated on single trial-blocks (one repetition of eight drifting grating directions per eye), trial-block 1 versus trial-block 9. Black: Real data. Gray: ODI's shuffled across neurons. Data of mouse M02. **b,** ODI maps based on single trial-block ODI values. Black circles: Identified ipsi-clusters. Scale bar: 100 µm. **c,** Identified ipsi-clusters across individual trial-blocks (note that some clusters are not clearly visible as they overlay with each other). **d-f,** As (**a**-**c**), but for mouse M05. **g,** Left: ODI as a function of distance to ipsi-cluster centers for trial-blocks 1 to 5. Black: Mean ± s.e.m., gray: Individual mice (n = 9). Right: Mean ODI inside ("In", 0-100 µm) and outside ("Out", 100-200 µm) ipsi-clusters (two-sided WMPSR test, W = 0, p = 0.004, n = 9 mice). **h,** As (**g**), but for trial-blocks 6 to 10 (two-sided WMPSR test, W = 0, p = 0.004, n = 9 mice). **i,** The standard deviation of the ODI value across individual trial-blocks of each neuron, as a function of distance of that neuron to ipsi-cluster centers (black line shows mean ± s.e.m. across mice; two-sided WMPSR test, p > 0.05, n = 9 mice).

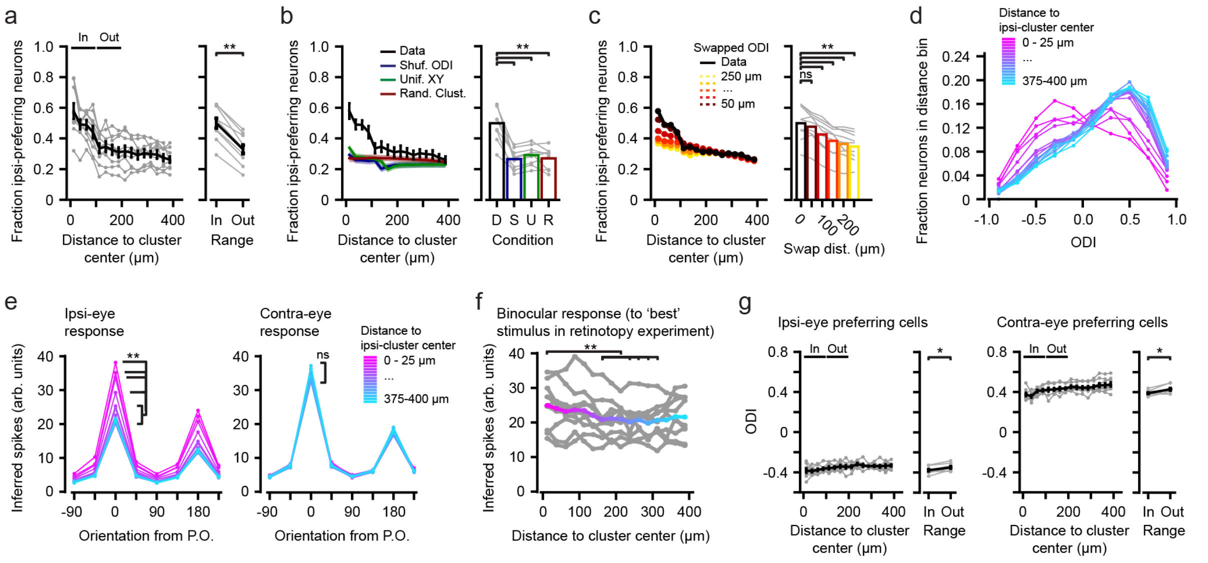

Supplementary Figure 4 | Fraction of ipsi-preferring neurons, response amplitude and ODI relative to ipsi-clusters.

a-c, Fraction (mean ± s.e.m.) of ipsi-preferring neurons as a function of distance to ipsi-clusters. **a,** Comparison of ODI "In" (100 µm range) and "Out" (100-200 µm) of ipsi-cluster centers (two-sided WMPSR test, W = 0, p = 0.004, n = 9 mice). **b,** Comparison of original data with global randomization controls, shuffled ODI values (blue), random XY positions (green) and randomly sampled cluster positions (red; two-sided Kruskal-Wallis test, H_3 = 16.3, p = 0.001, post hoc two-sided WMPSR test, ** p < 0.01, n = 9 mice). **c,** As (**b**), but for local randomization control (swapping ODIs of pairs of neurons; two-sided Kruskal-Wallis test, H_5 = 14.2, p = 0.015, post hoc two-sided WMPSR test, ns not significant, * p < 0.05, ** p < 0.01, n = 9 mice). **d,** Histogram of ODI as a function of distance of cells to ipsi-cluster centers. **e,** Response amplitude to ipsilateral (left) and contralateral eye (right) stimulation, averaged across all neurons, aligned to the preferred direction and as a function of distance to ipsi-clusters (see color legend). Ipsilateral eye stimulation: two-sided Kruskal-Wallis test, H_{15} = 98.5, p = $2.5\cdot10^{-14}$, post hoc two-sided WMPSR test, ** p < 0.01; Contralateral eye stimulation: two-sided Kruskal-Wallis test, H_{15} = 18.0, p = 0.26, n = 9 mice. **f,** Response amplitude (mean ± s.e.m.) to binocular stimulation (best stimulus in retinotopic mapping experiment) as function of distance to ipsi-clusters (two-sided Kruskal-Wallis test, H_{15} = 45.2, p = $7.2\cdot10^{-5}$, post hoc two-sided WMPSR test, ** p < 0.01; n = 9 mice). **g,** ODI as function of distance to ipsi-cluster centers, separately for ipsilateral and contralateral eye preferring neurons (Ipsi: two-sided WMPSR test, W = 4, p = 0.027; Contra: two-sided WMPSR test, W = 3, p = 0.020; n = 9 mice).

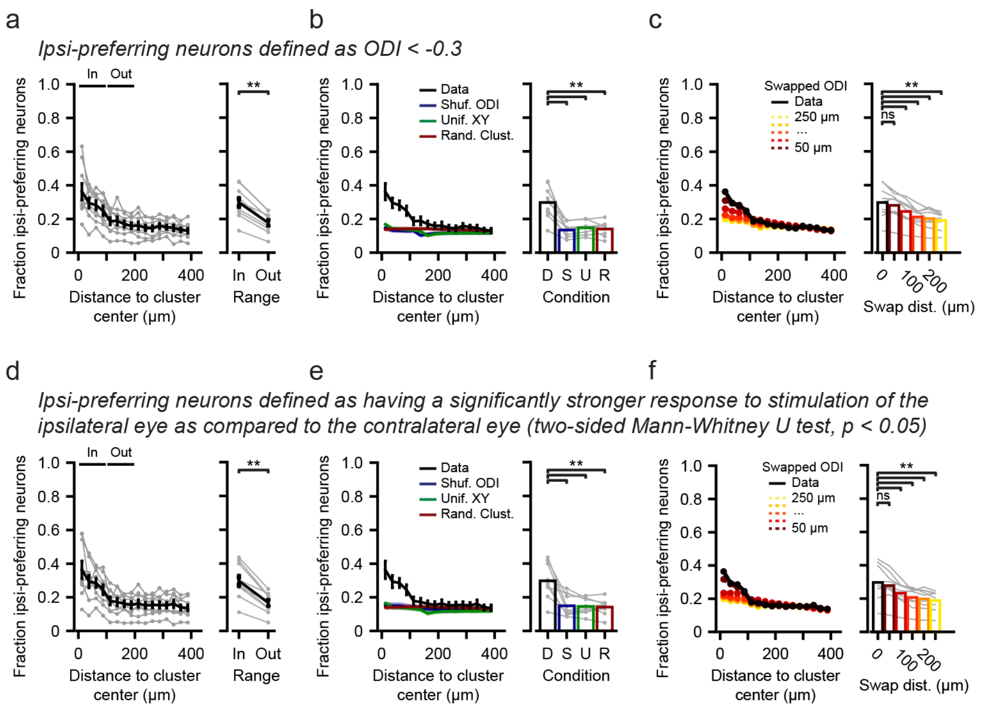

Supplementary Figure 5 | Fraction of ipsi-preferring neurons relative to ipsi-cluster centers, calculated using different definitions of 'ipsi-preferring'.

a-c, Fraction of ipsi-preferring neurons (defined as having an ODI smaller than -0.3) as a function of distance to ipsi-clusters; see also Supplementary Fig. 4a-c. **a,** Comparison of ODI "In" (100 µm range) and "Out" (100-200 µm) of ipsi-cluster centers. **b,** Comparison of original data with global randomization controls, shuffled ODI values (blue), random XY positions (green) and randomly sampled cluster positions (red). **c,** As (**b**), but for local randomization controls (swapping ODIs of pairs of neurons). **d-f**, As (**a**-**c**), but for ipsi-preferring neurons defined as having a significantly stronger response to the ipsilateral eye as compared to the contralateral eye. All panels: ns not significant, ** $p < 0.01$, $n = 9$ mice.

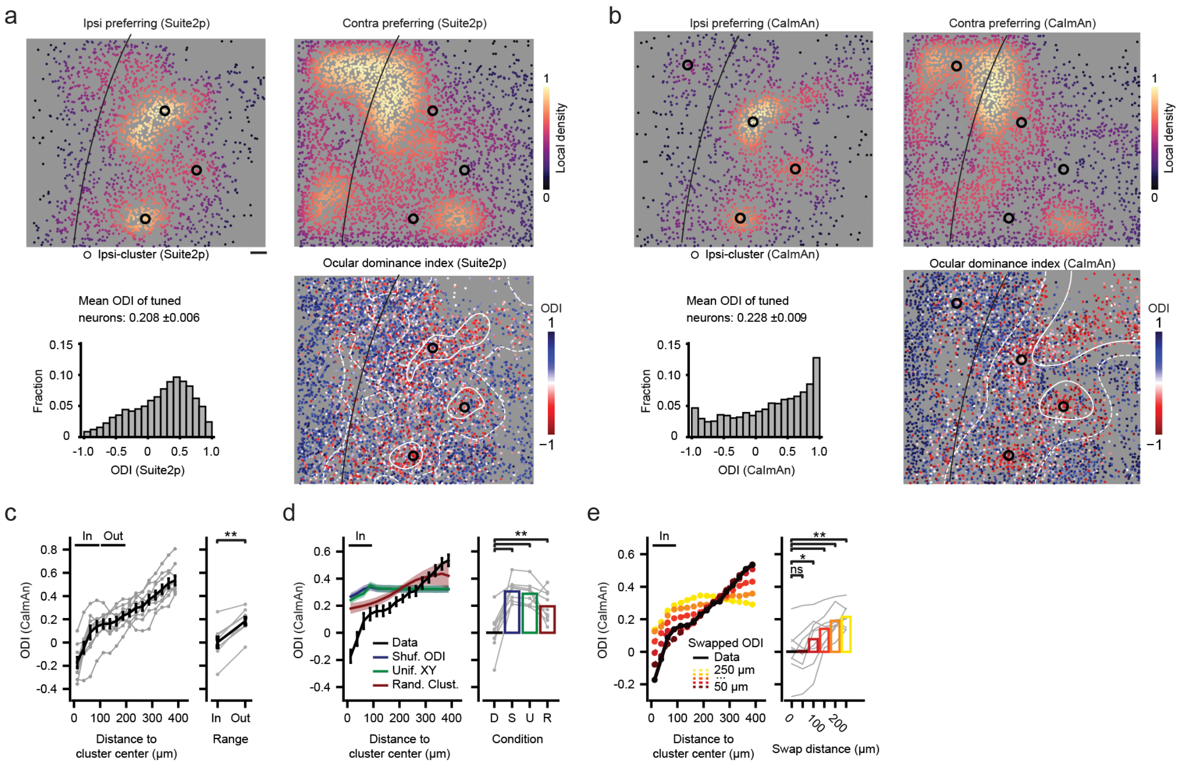

Supplementary Figure 6 | Comparison across preprocessing methods.

a, Density of ipsilateral and contralateral eye preferring neurons, a histogram of ODI values and a map showing the spatial distribution of ODI values across cortical layer 4 (same data as in Fig. 1c and d; mouse M02; Suite2p-based preprocessing and ROI extraction). Scale bar: 100 µm. **b,** Same as (**a**), but for CaImAn-based preprocessing and ROI extraction. **c-e**, As Fig. 1g-i, but for CaImAn-based processing ("In" vs. "Out": Two-sided WMPSR test, W = 0, p = 0.004; Global randomization controls: Two-sided Kruskal-Wallis test, H_3 = 18.4, $p = 3.6\cdot10^{-4}$, post hoc two-sided WMPSR test, ** p < 0.01; Local randomization control: Two-sided Kruskal-Wallis test, H_5 = 20.5, p = 0.001, post hoc two-sided WMPSR test, ns not significant, * p < 0.05, ** p < 0.01; n = 9 mice).

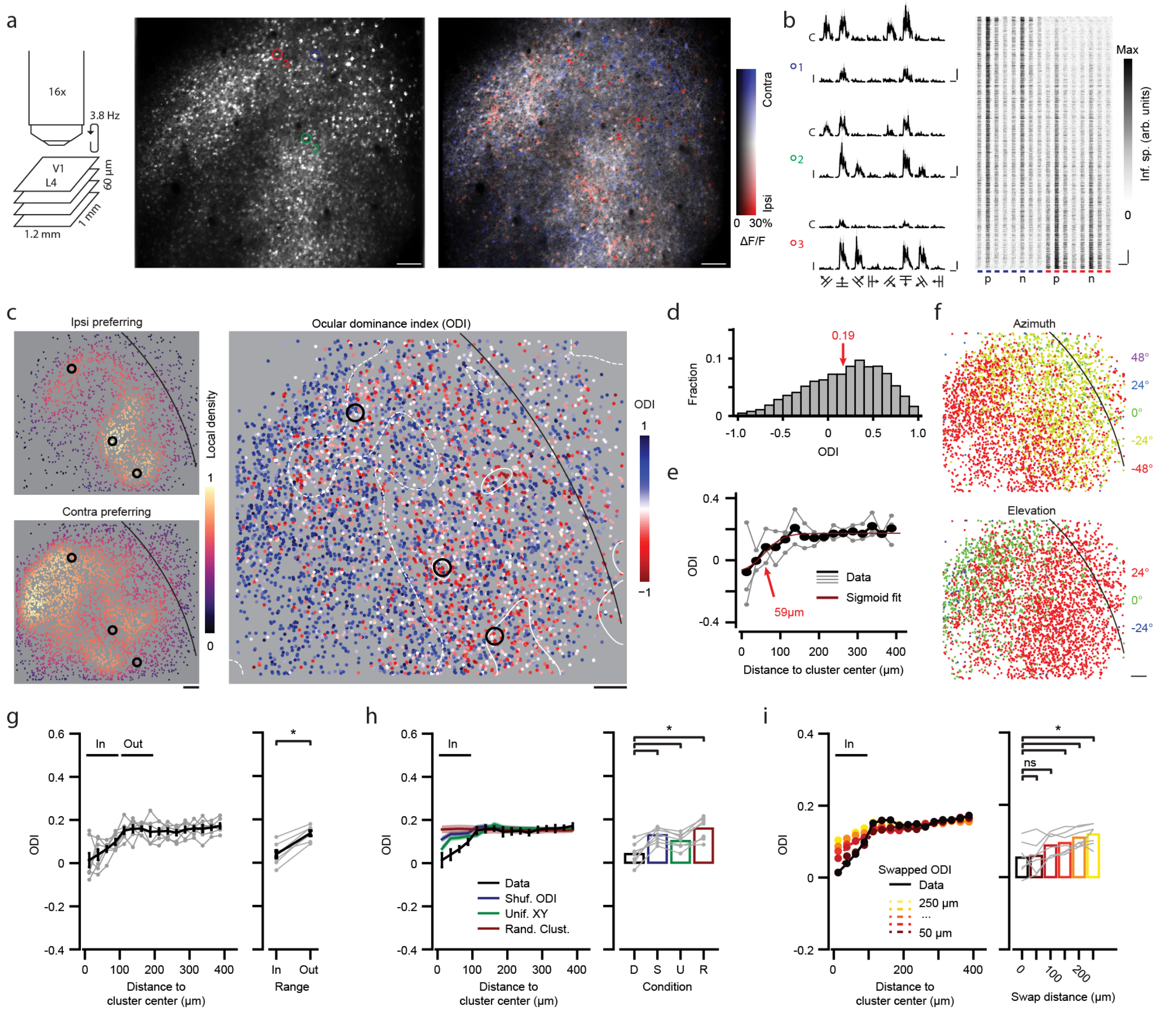

Supplementary Figure 7 | Layer 4 ipsilateral eye preferring ocular dominance clusters in Scnn1a-Tg3-Cre transgenic mice expressing the calcium indicator jRGECO1a via AAV delivery.

As Fig. 1, based on imaging the red-fluorescent calcium indicator jRGECO1a in Scnn1a-Tg3-Cre mice. Using an AAV, the indicator was conditionally expressed in right-hemisphere L4 neurons. Note that because these data were obtained from the right hemisphere, the maps appear flipped along the horizontal axis compared to e.g. Fig. 1. **a,** Left: Schematic of multilevel acquisition and field of view dimensions. Middle: Projection of an example motion corrected imaging stack (single plane of a four-plane volume) showing neurons expressing jRGECO1a. Right: HLS map showing eye selective responses during visual stimulation (Hue: Preferred eye. Lightness: ΔF/F response amplitude. Saturation: Eye selectivity. Scale bar: 100 µm. **b,** Left: Trial-averaged peri-stimulus ΔF/F responses to eight directions and two eyes, for three example neurons (colored circles in **a**). Right: Trial-averaged inferred spiking responses of all visually responsive neurons (n = 1794) in the imaging plane shown in (**a**). **c,** Left: Local density of ipsilateral (top) and contralateral (bottom) eye preferring visually responsive neurons across the (four-plane) volume of the example mouse (M10). Black circles indicate detected ipsi-cluster centers. The black line marks the higher area boundary between V1 (left) and lateral higher visual areas (right). Right: ODI of all visually responsive neurons. White lines indicate ODI = 0 (solid) and ODI = 0.2 (dashed). Scale bar: 100 µm. **d,** Histogram showing the distribution of ODI values across the example volume. **e,** ODI as function of distance to detected ipsi-cluster centers for the example volume (Gray: individual centers. Black: Mean. Red: Sigmoid fit. Arrow: Point of maximum inclination, approximates the ipsi-cluster radius). **f,** Preferred azimuth (top) and elevation (bottom) of all visually responsive neurons in the example volume. Black line separates V1 (left) from lateral higher areas (right). Scale bar: 100 µm. **g,** Left: Mean (±s.e.m.) ODI as function of distance to ipsi-cluster centers (Gray: individual mice). Right: ODI within a 100 µm range ("In") of ipsi-cluster centers, compared to outside that range ("Out", 100 µm-200 µm; two-sided WMPSR test, W = 0, p = 0.016, n = 7 mice). **h,** Left: As (**g**), but comparing original data with global randomization controls, shuffling ODI values across neurons (blue), assigning neurons new XY positions randomly sampled from a uniform distribution (green) and assigning new ipsi-cluster centers by random sampling (red). Right: Quantification, showing mean within ipsi-cluster ODI for each condition (two-sided

Kruskal-Wallis test, $H_3 = 16.6$, $p = 0.009$, post hoc two-sided WMPSR test, * $p < 0.05$, $n = 7$ mice). **i,** As (**g**), but for local randomization control, swapping the ODIs of pairs of neurons spaced apart 50 µm (dark red) to 250 µm (yellow; two-sided Kruskal-Wallis test, $H_5 = 14.0$, $p = 0.016$, post hoc two-sided WMPSR test, ns not significant, * $p < 0.05$, $n = 7$ mice).

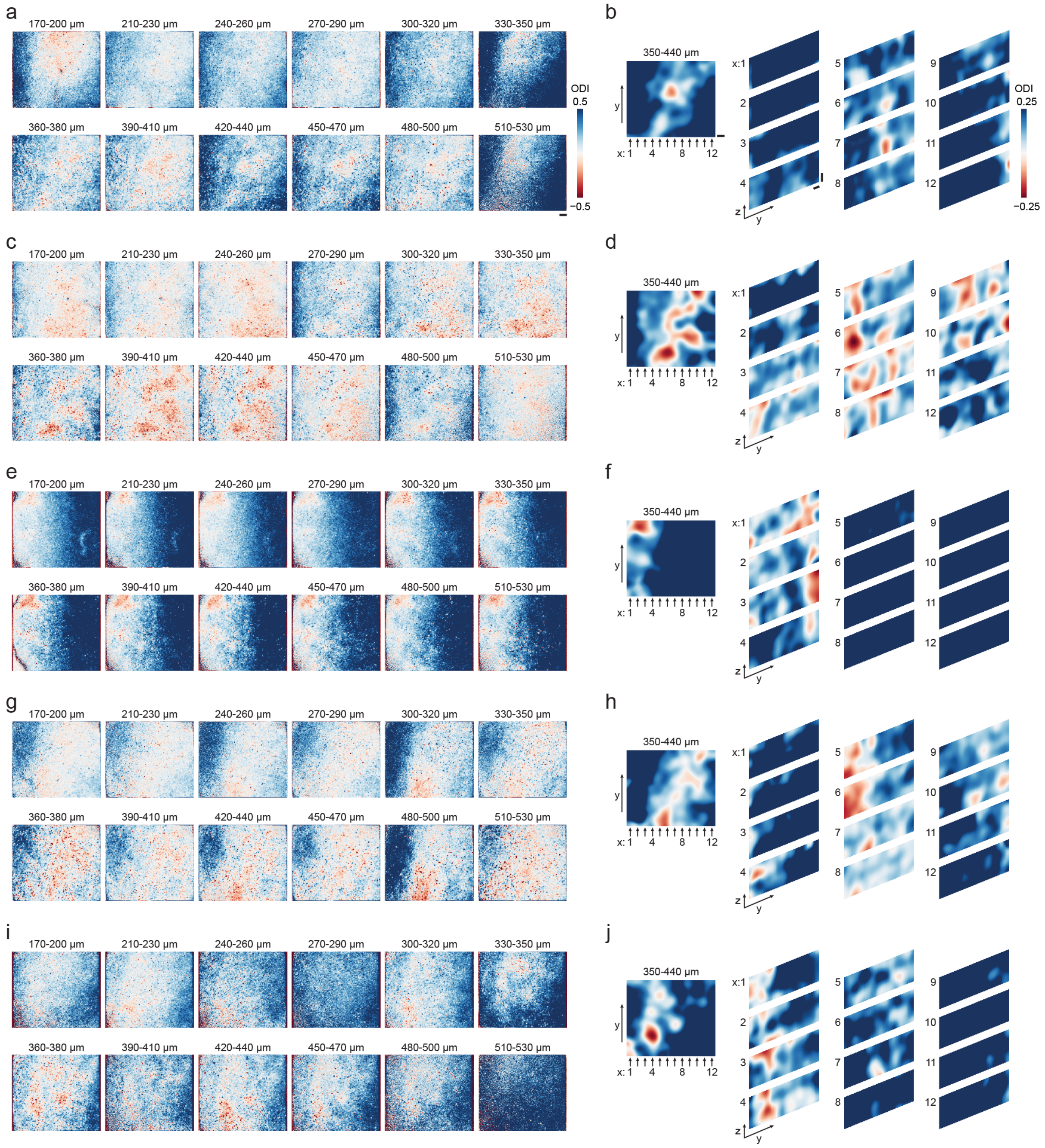

Supplementary Figure 8 | Examples of ocular dominance patterns spanning cortical layers.
a, Pixelwise ODI maps calculated on small sub-volumes of imaging data, acquired in multiple consecutive four-plane volumes across a total depth range of 360 µm (170-530 µm below cortical surface; see Fig. 2a for schematic). Scale bar: 100 µm. **b,** Left: Smoothed pixelwise ODI map of cortical layer 4 (data from 350-440 µm below cortical surface). Arrows indicate the direction along which the three-dimensional imaged volume is re-sliced to generate the side-view, smoothed ODI maps on the right. Right: Vertical slices showing smoothed pixel ODI maps spanning L2/3 to upper L5. Scale bar: 100 µm. **a-j,** Each row represents data from a single example mouse: **a-b**, M01. **c-d**, M02. **e-f**, M03, **g-h**, M05, **i-j**, M06.

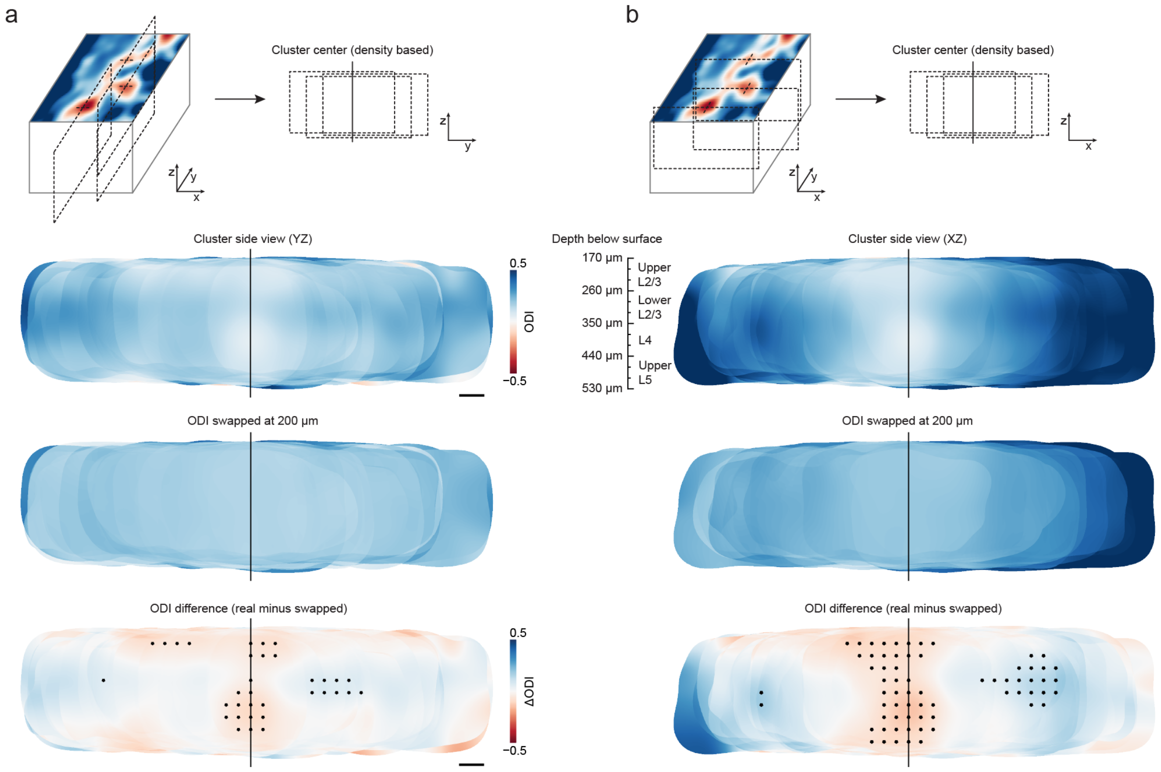

Supplementary Figure 9 | ODI maps in upper and lower cortical layers relative to layer 4 ipsi-cluster centers. **a,** ODI maps of vertically oriented slices (anterior-posterior), center-aligned at identified ipsi-clusters. Top: Schematic of vertical slice alignment. Middle: The mean (across mice) of all cluster-aligned vertical slices, with real data first and locally shuffled data below (swapped ODIs at 200 µm). Bottom: Difference between real and shuffled data (two-sided WMPSR test, * $p < 0.05$, $n = 9$ mice). All side views: The black vertical line indicates the center (origin) of the side-view, to which ipsi-clusters were aligned. Scale bar: 100 µm. **b,** As (**a**), but for slices along the medial-lateral orientation).

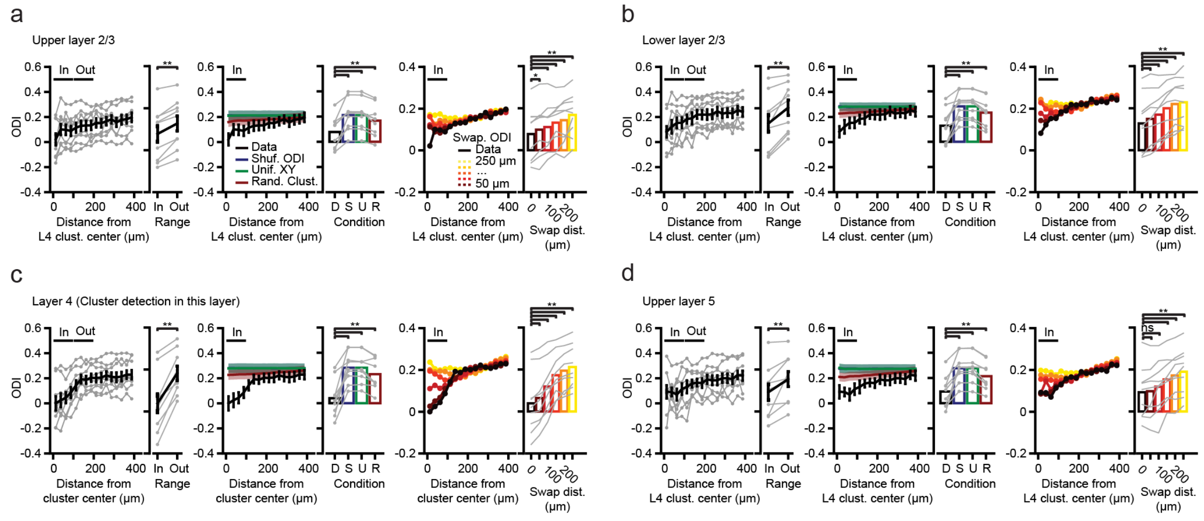

Supplementary Figure 10 | ODI of neurons in upper and lower cortical layers as a function of distance to ipsi-cluster centers identified in layer 4.

a-d, As Fig. 1g-i, for four sub-volumes spanning the depth range from 170 to 530 µm below cortical surface. Mean (±s.e.m.) ODI of upper L2/3 neurons as function of distance to L4-identified ipsi-cluster centers comparing “In” vs. “Out”, global randomization controls and the local randomization control. Testing, per sub-volume, ODI “In” ipsi-cluster centers against all controls, i.e. “Out”, global and local randomization, normalized per mouse. **a,** Upper layer 2/3: Two-sided Kruskal-Wallis test, H_9 = 57.2, $p = 4.5 \cdot 10^{-8}$, post hoc two-sided WMPSR test, * $p < 0.05$, ** $p < 0.01$, n = 9 mice. **b,** Lower layer 2/3: Two-sided Kruskal-Wallis test, H_9 = 47.9, $p = 2.7 \cdot 10^{-6}$, post hoc two-sided WMPSR test, ** $p < 0.01$, n = 9 mice. **c,** Layer 4: Two-sided Kruskal-Wallis test, H_9 = 55.9, $p = 8.3 \cdot 10^{-8}$, post hoc two-sided WMPSR test, ** $p < 0.01$, n = 9 mice. **d,** Upper layer 5: Two-sided Kruskal-Wallis test, H_9 = 56.3, $p = 7.0 \cdot 10^{-8}$, post hoc two-sided WMPSR test, ns not significant, ** $p < 0.01$, n = 9 mice.

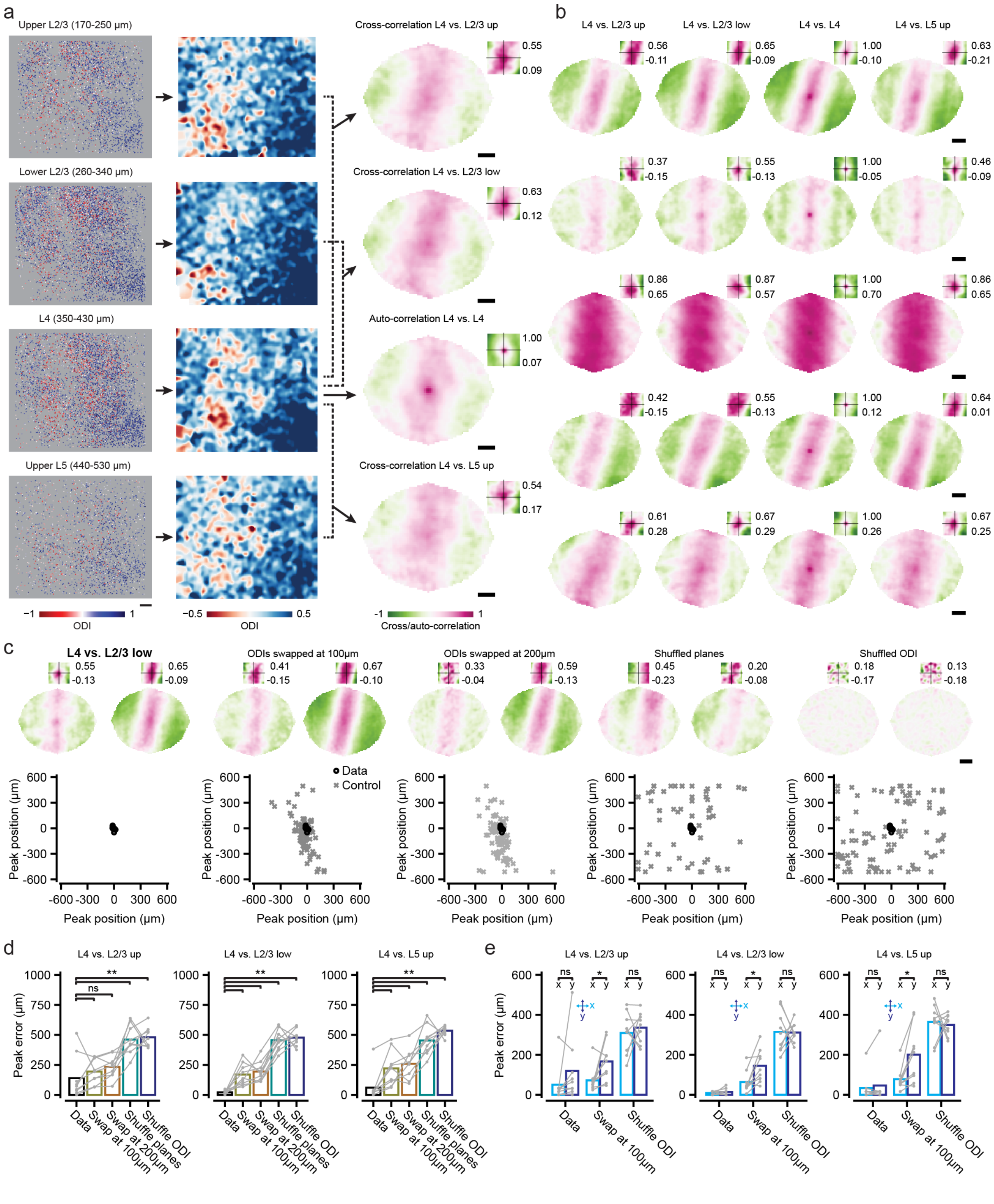

Supplementary Figure 11 | Cross-correlation analysis of ODI patterns across cortical layers reveals a local and global organization for ocular dominance.

a, Left: For one example mouse (M06), the ODI of all visually responsive neurons in each of four depth-ranges spanning the sampled cortical volume from L2/3 to L5 (see Fig. 2a). Middle: Smoothed ODI maps (see Methods). Right: Auto-/cross-correlation of each ODI map with the L4 ODI map. Right-top: Insets show the cropped map-center of each cross/auto-correlation map, scaled to its respective minimum and maximum correlation value (indicated next to the inset). The scale of the inset is identical to the adjacent cross-correlation map. Scale bar, left: 100 µm. Scale bar, right: 200 µm. **b,** Each row shows the four auto-/cross-correlation maps (as in **a**) of five further example mice (from top to bottom: M01, M02, M03, M05, M04). Scale bar: 200 µm. **c,** Left column, original data. Remaining columns: Local and global randomization controls (ODIs swapped at 100 µm, 200 µm: ODIs of random pairs of cells at approximately a distance of 100 µm or 200 µm were swapped; shuffled planes: Cross-correlation maps calculated between ODI maps of different animals; shuffled ODI: Cross-correlation maps calculated using ODI maps from data with shuffled ODI values; 10 shuffled datasets per mouse). For each column,

top: Cross-correlation maps from two example mice (M02 and M01). Bottom: XY coordinates of all map peaks (original data, n = 9 mice, black; shuffled data, n = 9 mice, 10 shuffles per mouse, gray). Scale bar: 200 µm. **d,** Peak error, defined as the Euclidian distance of the cross-correlation map peak to the center of the map, for original data and local and global randomization controls (see **c**; L2/3 up: Two-sided Kruskal-Wallis test, $H_4 = 27.8$, $p = 1.4 \cdot 10^{-5}$; L2/3 low: Two-sided Kruskal-Wallis test, $H_4 = 37.8$, $p = 1.2 \cdot 10^{-7}$; L5 up: Two-sided Kruskal-Wallis test, $H_4 = 32.0$, $p = 1.9 \cdot 10^{-6}$; post hoc two-sided WMPSR tests, ns not significant, ** $p < 0.01$, n = 9 mice). **e,** As (**d**), but for the peak error along the horizontal and vertical dimensions of the cross-correlation maps separately (L2/3 up: Two-sided Kruskal-Wallis test, $H_4 = 32.1$, $p = 5.7 \cdot 10^{-6}$; L2/3 low: Two-sided Kruskal-Wallis test, $H_4 = 45.5$, $p = 1.1 \cdot 10^{-8}$; L5 up: Two-sided Kruskal-Wallis test, $H_4 = 38.9$, $p = 2.5 \cdot 10^{-7}$; post hoc two-sided WMPSR tests, ns not significant, * $p < 0.05$, n = 9 mice). **d,e,** Bars show mean across mice.

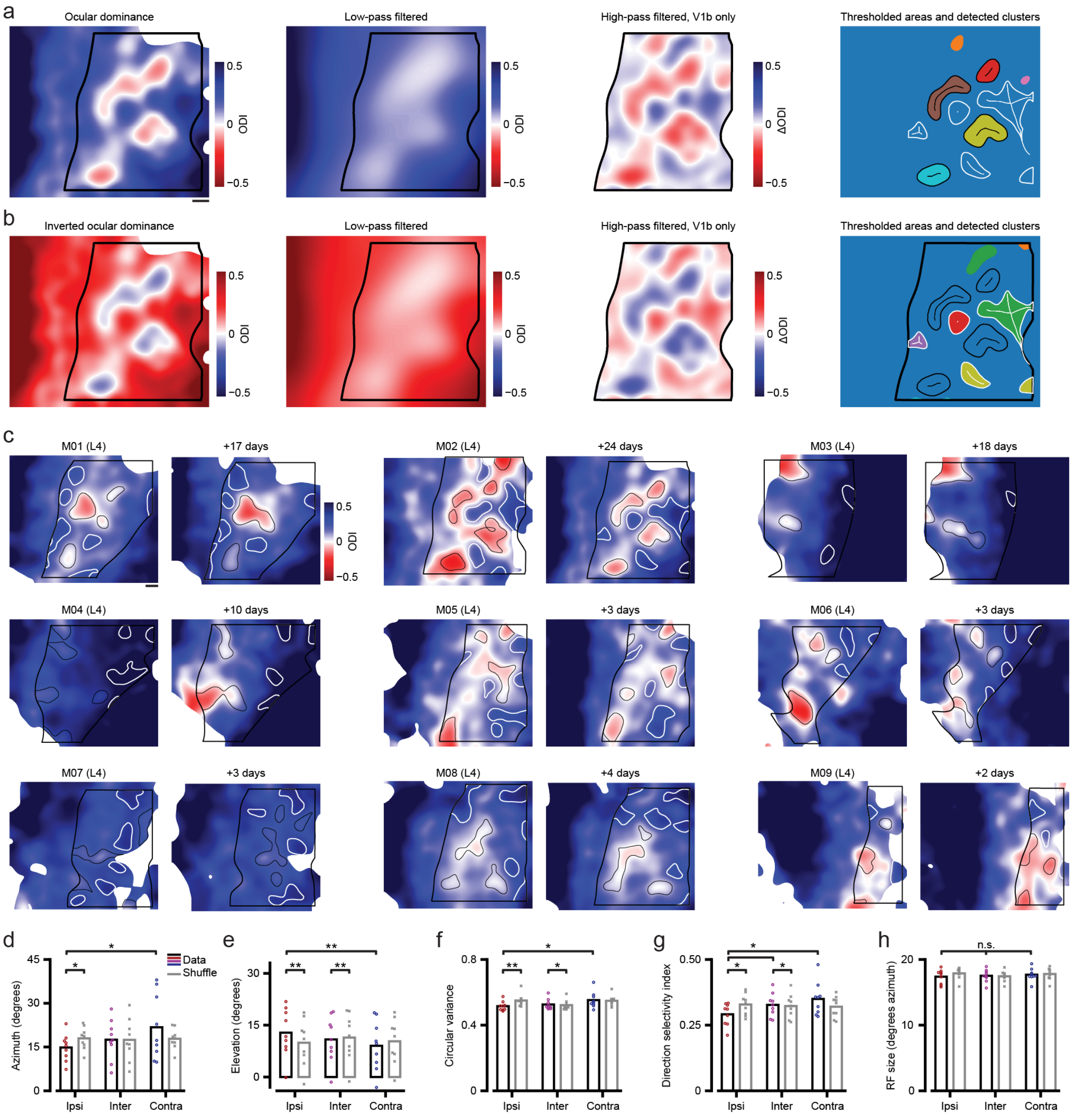

Supplementary Figure 12 | Identification of geometrically defined clusters from smoothed ODI maps

a,b, Steps for identifying geometrically defined clusters from ODI maps, from left to right: A smoothed ODI map (Gaussian, σ = 42 µm); a low pass ODI map (Gaussian, σ = 105 µm); a high pass ODI map (smoothed map minus low pass map), highlighting local increases/decreases in ODI; a thresholded map (high-pass ODI < 15th percentile) with boundaries and skeletons for areas within the valid V1b region that have (1) a diameter > 100 µm and (2) an average ODI < 25th percentile of the V1b ODI map. **a,** Ipsilateral eye preferring regions. **b,** Contralateral eye preferring regions. Scale bar: 100 µm. **c,** For each mouse, pairs of ODI maps imaged 2 to 24 days apart, showing V1b (thick black outlined region) and geometry-identified ipsi- (black lines) and contra- (white lines) regions. Scale bar: 100 µm. **d,** Preferred azimuth of neurons in ipsi- (red), contra- (blue) and inter-cluster (pink) regions (Ipsi, inter, contra: Two-sided Kruskal-Wallis test, H_2 = 8.9, p = 0.012, post hoc two-sided WMPSR test, * $p < 0.05$; Two-sided WMPSR test; Ipsi vs. shuffle: W = 4, p = 0.027; n = 9 mice). **e,** As (**d**), for Elevation (Ipsi, inter, contra: Two-sided Kruskal-Wallis test, H_2 = 15.4, p = 0.0005, post hoc two-sided WMPSR test, ** $p < 0.01$; Two-sided WMPSR test; Ipsi vs. shuffle: W = 1, p = 0.008; Inter vs. shuffle: W = 0, p = 0.004; n = 9 mice). **f,** As (**d**), for Circular variance (Ipsi, inter, contra: Two-sided Kruskal-Wallis test, H_2 = 12.8, p = 0.0017, post hoc two-sided WMPSR test, * $p <$ 0.05; Two-sided WMPSR test; Ipsi vs. shuffle: W = 1, p = 0.008; Inter vs. shuffle: W = 2, p = 0.012; n = 9 mice). **g,** As (**d**), for direction selectivity index (Ipsi, inter, contra: Two-sided Kruskal-Wallis test, H_2 = 11.8, p = 0.0028, post hoc two-sided WMPSR test, * $p < 0.05$; Two-sided WMPSR test; Ipsi vs. shuffle: W = 2, p = 0.012; Inter vs. shuffle: W = 3, p = 0.020; n = 9 mice). **h,** As (**d**), for receptive field size along the azimuth axis (Ipsi, inter, contra: Two-sided Kruskal-Wallis test, $p > 0.05$; n = 9 mice). **d-h,** Bars show mean across mice.

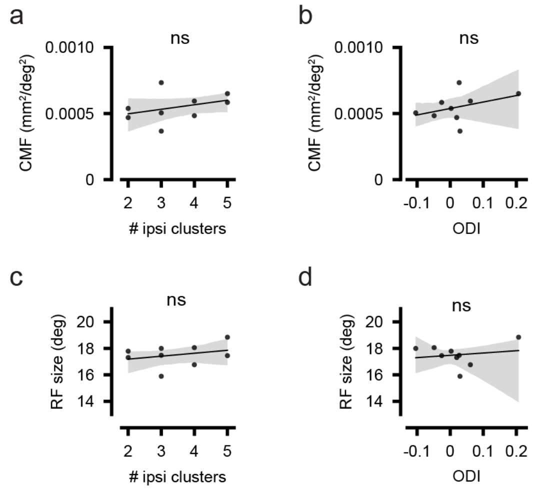

Supplementary Figure 13 | Correlation of descriptive variables indicating 'ipsi-cluster quality', with the cortical magnification factor and neuronal receptive field size.

a, Cortical magnification factor (CMF) in $mm^2/degree^2$ (mm^2/deg^2) versus the number of ipsi-clusters in each mouse. **b,** As (**a**), but for the mean ODI in ipsi-clusters. **c,** Receptive field (RF) size, in degrees (deg) azimuth, versus the number of ipsi-clusters in each mouse. **d,** As (**c**), but for the ODI in ipsi-clusters. All panels: Individual black dots represent mice (n = 9). The regression line shows the best linear fit, with shaded regions indicating the 95% confidence interval (ns not significant: Pearson correlation coefficient, $p > 0.05$).

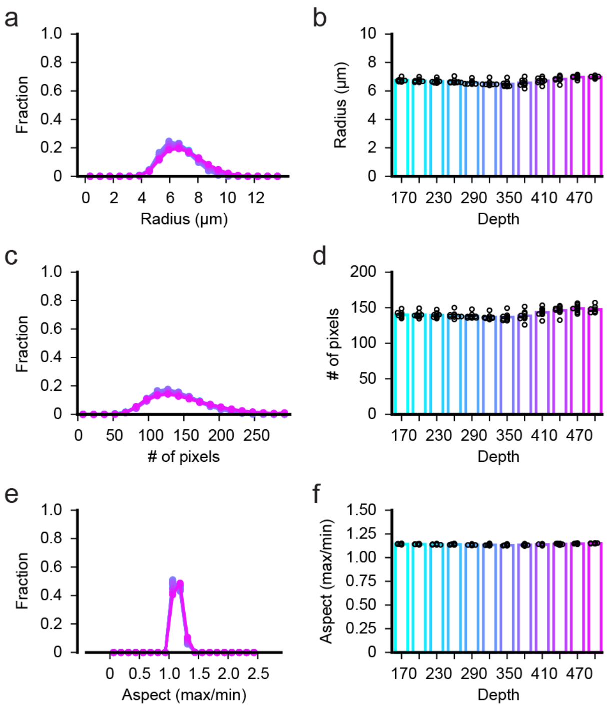

Supplementary Figure 14 | Shape quantification of ROI footprints across cortical layers.
a, Distributions of ROI footprint radii across cortical depths (for depth-color legend, see bars in **b**). Note that several distribution curves are not visible as they are very similar to the foreground curve. **b,** Mean radius for each depth range. **c,d,** As (**a**,**b**), but for the area of the ROI footprints (expressed in number of pixels). **e,f,** As (**a**,**b**), but for the aspect ratio of the ROI footprints.
