# Supplementary material for: A column-like organization for ocular dominance in mouse visual cortex
Source: Nat Commun. 2025 Feb 25;16:1926. doi: 10.1038/s41467-025-56780-3 (PMC11861588; doi:10.1038/s41467-025-56780-3)
Supplement: Supplementary file 2 — Description of Additional Supplementary Files [file 41467_2025_56780_MOESM2_ESM.pdf]

Description of Additional Supplementary Files

File Name: Supplementary Data 1
Description: Tabularized data (.xls) listing 43 parameters describing properties and interactions of recorded ipsi-clusters (tab 1). Tabs 2 to 4 hold the same information for contralateral eye preferring regions, regions between ipsi-clusters and contralateral eye dominated regions, and regions corresponding to the imaged part of binocular V1. Tab 5 describes the variables in more detail and Tab 6 lists the sex of individual mice.
