# Supplementary material for: A column-like organization for ocular dominance in mouse visual cortex
Source: Nat Commun. 2025 Feb 25;16:1926. doi: 10.1038/s41467-025-56780-3 (PMC11861588; doi:10.1038/s41467-025-56780-3)
Supplement: Supplementary file 4 — Reporting Summary [file 41467_2025_56780_MOESM4_ESM.pdf]

nature portfolio

Corresponding author(s): Pieter Goltstein
Mark Hübener

Last updated by author(s): 8 Jan 2024

Reporting Summary

Nature Portfolio wishes to improve the reproducibility of the work that we publish. This form provides structure for consistency and transparency in reporting. For further information on Nature Portfolio policies, see our Editorial Policies and the Editorial Policy Checklist.

Statistics

For all statistical analyses, confirm that the following items are present in the figure legend, table legend, main text, or Methods section.

n/a	Confirmed	
☐	☒	The exact sample size (n) for each experimental group/condition, given as a discrete number and unit of measurement
☐	☒	A statement on whether measurements were taken from distinct samples or whether the same sample was measured repeatedly
☐	☒	The statistical test(s) used AND whether they are one- or two-sided<br>*Only common tests should be described solely by name; describe more complex techniques in the Methods section.*
☐	☒	A description of all covariates tested
☐	☒	A description of any assumptions or corrections, such as tests of normality and adjustment for multiple comparisons
☐	☒	A full description of the statistical parameters including central tendency (e.g. means) or other basic estimates (e.g. regression coefficient) AND variation (e.g. standard deviation) or associated estimates of uncertainty (e.g. confidence intervals)
☐	☒	For null hypothesis testing, the test statistic (e.g. F, t, r) with confidence intervals, effect sizes, degrees of freedom and P value noted<br>*Give P values as exact values whenever suitable.*
☒	☐	For Bayesian analysis, information on the choice of priors and Markov chain Monte Carlo settings
☒	☐	For hierarchical and complex designs, identification of the appropriate level for tests and full reporting of outcomes
☐	☒	Estimates of effect sizes (e.g. Cohen's d, Pearson's r), indicating how they were calculated

Our web collection on statistics for biologists contains articles on many of the points above.

Software and code

Policy information about availability of computer code

Data collection	Data were collected using Matlab versions 2013 and 2014, and LabView version 2014. The software 'Scanimage version 4' (Vidrio Technologies) was used to acquire two-photon microscopy data. Visual stimuli were presented using Psychophysics Toolbox extensions (version 3). Other software was custom written and will be shared upon request, noting that this is not production environment ready code and will require knowledge of the Matlab and LabView platforms.
Data analysis	Data analysis was done using Matlab version 2017 and Python versions 3.8.12, 3.8.17, 3.10.8, 3.12.3 (using Numpy versions 1.20.3, 1.21.5, 1.26.0, 1.26.4, Scipy versions 1.7.3, 1.10.1, 1.11.3, 1.13.0, Suite2P versions 0.7, 0.10.3, and CaImAn version 1.9.16). Custom written Matlab and Python routines used for data collection and data preprocessing are available upon request. The Python code used for data analysis and producing figures available on https://github.com/pgoltstein/mouse-od-columns/.

For manuscripts utilizing custom algorithms or software that are central to the research but not yet described in published literature, software must be made available to editors and reviewers. We strongly encourage code deposition in a community repository (e.g. GitHub). See the Nature Portfolio guidelines for submitting code & software for further information.

Data

Policy information about availability of data

All manuscripts must include a data availability statement. This statement should provide the following information, where applicable:

- Accession codes, unique identifiers, or web links for publicly available datasets
- A description of any restrictions on data availability
- For clinical datasets or third party data, please ensure that the statement adheres to our policy

The data generated in this study are publicly available on https://gin.g-node.org/pgoltstein/mouse-od-columns/.

Research involving human participants, their data, or biological material

Policy information about studies with human participants or human data. See also policy information about sex, gender (identity/presentation), and sexual orientation and race, ethnicity and racism.

Reporting on sex and gender	n/a
Reporting on race, ethnicity, or other socially relevant groupings	n/a
Population characteristics	n/a
Recruitment	n/a
Ethics oversight	n/a

Note that full information on the approval of the study protocol must also be provided in the manuscript.

Field-specific reporting

Please select the one below that is the best fit for your research. If you are not sure, read the appropriate sections before making your selection.

☒ Life sciences ☐ Behavioural & social sciences ☐ Ecological, evolutionary & environmental sciences

For a reference copy of the document with all sections, see nature.com/documents/nr-reporting-summary-flat.pdf

Life sciences study design

All studies must disclose on these points even when the disclosure is negative.

Sample size	We did not use statistical methods to predetermine the sample size, but used an n similar to that reported in previous publications (see e.g. Mrsic-Flogel et al., Neuron, 2007 and Rose et al., Science, 2016).
Data exclusions	We excluded data of one Scnn1a-Tg3-Cre transgenic mouse (of the dataset shown in Supplementary Fig. 7) because the signal-to-noise ratio was low and Suite2P only detected ~300 neurons in the entire L4 volume, which was not sufficient for identifying densities of ipsilateral eye preferring neurons.
Replication	We replicated the main finding of our study in a different transgenic mouse line (i.e. compare Fig. 1 with Supplementary Fig. 7).
Randomization	There was no assignment of animals into experimental groups. Stimulus presentations were randomized within and across blocks, as described in the Methods subsection "Imaging". Methods for randomized control analyses are described in the Methods subsection "Data analysis: Randomization procedures".
Blinding	Experimenters were not blinded to experimental conditions or group allocation. This is because the study does not explicitly define or compare experimental groups, but instead aims to provide an objective description of the functional organization of neurons in the mouse visual cortex.

Reporting for specific materials, systems and methods

We require information from authors about some types of materials, experimental systems and methods used in many studies. Here, indicate whether each material, system or method listed is relevant to your study. If you are not sure if a list item applies to your research, read the appropriate section before selecting a response.

Materials & experimental systems

n/a	Involved in the study
☒	☐ Antibodies
☒	☐ Eukaryotic cell lines
☒	☐ Palaeontology and archaeology
☐	☒ Animals and other organisms
☒	☐ Clinical data
☒	☐ Dual use research of concern
☒	☐ Plants

Methods

n/a	Involved in the study
☒	☐ ChIP-seq
☒	☐ Flow cytometry
☒	☐ MRI-based neuroimaging

Animals and other research organisms

Policy information about studies involving animals; ARRIVE guidelines recommended for reporting animal research, and Sex and Gender in Research

Laboratory animals	We used nine adult mice (6 female, 3 male; four to five months of age during data acquisition) genetically expressing the calcium indicator GCaMP6s in excitatory neurons (B6;DBA-Tg(tetO-GCaMP6s)2Niell/J24, JAX stock #024742; back-crossed for at least seven generations to C57Bl/6NRj) crossed with B6.Cg-Tg(Camk2a-tTA)1Mmay/DboJ73 (JAX stock #007004; maintained on a mixed background of C57BL/6NRj and C57BL/6J) and seven adult Scnn1a-Tg3-Cre mice (4 female, 3 male; four months of age during data acquisition; B6;C3-Tg(Scnn1a-cre)3Aibs/J30, JAX stock #009613; kept on a mixed background of C57BL/6 and C3H). The animals were housed in small groups, or individually in case of inter-male aggression, in large cages (GR900, Tecniplast) containing a running wheel, a tunnel and a shelter. The animals were kept on a reversed day/night cycle with lights on at 22:00 h and lights off at 10:00 h. Ambient temperature (21.0 ±0.7°C) and humidity (63 ±2%) were kept constant. Food and water were available ad libitum.
Wild animals	No wild animals were used in this study.
Reporting on sex	Findings were confirmed in both female and male laboratory mice in the main experiment (6 female, 3 male) and the replication (4 female, 3 male). Data on the sex of individual animals can be found in Supplementary Data 1.
Field-collected samples	No field-collected samples were used in this study.
Ethics oversight	All experiments were conducted following the institutional guidelines of the Max Planck Society and the regulations of the local government ethical committee (Beratende Ethikkommission nach §15 Tierschutzgesetz, Regierung von Oberbayern).

Note that full information on the approval of the study protocol must also be provided in the manuscript.

Plants

Seed stocks	n/a
Novel plant genotypes	n/a
Authentication	n/a
